# Prenatal Use of Sirolimus in Fetal Therapy: Current Evidence, Clinical Applications, and Future Directions

**DOI:** 10.7759/cureus.115909

**Published:** 2026-09-07

**Authors:** Zbigniew Ruszczak, Iwona Strzelecka, Maria Respondek-Liberska

**Affiliations:** 1 Department of Medicine and Dermatology, Khalifa University, Abu Dhabi, ARE; 2 Department of Diagnoses and Prevention of Fetal Malformations, Medical University of Lodz, Lodz, POL; 3 Department of Prenatal Cardiology, Polish Mother’s Memorial Hospital - Research Institute, Lodz, POL

**Keywords:** fetal cardiac rhabdomyoma, fetal safety, fetal tumour, lymphatic malformation, maternal, maternal safety, mechanistic target of rapamycin, transplacental treatment, tuberous sclerosis complex, vascular malformation

## Abstract

Prenatal imaging, genomic diagnosis, and pathway-directed pharmacology increasingly permit treatment of selected fetal disorders before birth. This narrative review evaluates maternally administered sirolimus as transplacental fetal therapy, emphasizing cardiac rhabdomyomas associated with tuberous sclerosis complex and severe vascular or lymphatic malformations. Sirolimus inhibits mechanistic target of rapamycin complex-1, a pathway central to cell growth, angiogenesis, and lymphangiogenesis.

Published fetal case reports and small series describe regression of symptomatic cardiac rhabdomyomas and improvement in outflow obstruction, arrhythmia, cardiac dysfunction, hydrops, or impending fetal compromise. Evidence for prenatal treatment of vascular and lymphatic malformations is earlier. Still, it includes large cervicofacial lymphatic malformations and complex capillary-lymphatic-venous malformations threatening airway obstruction, hemorrhage, hydrops, or perinatal instability. Reported maternal adverse effects include mucositis, hyperlipidemia, cytopenias, infection risk, and impaired wound healing. Transplant pregnancy data have not identified a consistent malformation pattern, but exposures are sparse and confounded, while product labeling warns of embryo-fetal toxicity based on animal studies.

Prenatal sirolimus therefore remains an investigational intervention for carefully selected, life-threatening or organ-threatening fetal disease managed by specialized multidisciplinary teams. Prospective registries and multicenter studies are needed to define dosing, fetal pharmacokinetics, stopping rules, delivery planning, and long-term outcomes.

## Introduction and background

Fetal therapy has entered an era in which prenatal imaging, genomic diagnosis, and targeted pharmacology can be combined to treat selected disorders before birth. Ultrasound and fetal echocardiography remain central to this process: they identify the evolving phenotype, quantify risk, guide timing of therapy, and provide objective biomarkers of response. Sirolimus, also known as rapamycin, is one of the most important candidates for transplacental targeted therapy because it acts on the PI3K-AKT-mTOR signaling axis, a pathway implicated in tuberous sclerosis complex, vascular anomalies, lymphatic malformations, diabetes, and even aging [1-4].

Sirolimus binds FKBP12 and inhibits mTORC1, reducing cellular growth, protein synthesis, angiogenesis, and lymphangiogenesis [1,2]. In non-pregnant populations, it is established in transplantation and has become an important therapy for complicated vascular anomalies [5-8].

In fetal medicine, the drug has moved from theoretical interest to clinical use through reports of maternal administration for symptomatic fetal cardiac rhabdomyomas and severe fetal lymphatic or combined vascular malformations [9-22].

The rationale for prenatal treatment is strongest when fetal disease is progressive, potentially lethal, and driven by a pathway likely to respond to mTOR inhibition. In fetal cardiac rhabdomyoma, especially in tuberous sclerosis complex (TSC), prenatal sirolimus may reduce tumor volume quickly enough to relieve outflow obstruction or cardiac failure before birth [9-19]. In fetal vascular anomalies, the therapeutic goal is not merely cosmetic regression but prevention of hydrops, hemorrhage, high-output failure, airway compromise, or death [20-22].

This review summarizes the evidence base for prenatal sirolimus therapy, proposes a practical ultrasound-centered treatment algorithm, and identifies priorities for future research. The article is intended as a clinically oriented review for fetal medicine specialists, obstetric sonologists, fetal cardiologists, geneticists, neonatologists, and multidisciplinary vascular anomaly teams.

## Review

Review methodology

This narrative review considered PubMed-indexed articles, publisher records, regulatory labeling, teratology resources, and reference metadata available through July 31, 2026. Evidence was selected for relevance to prenatal sirolimus exposure, fetal cardiac rhabdomyoma, tuberous sclerosis complex, vascular or lymphatic malformations, maternal safety, fetal pharmacology, or imaging-based monitoring. Case reports and small series were interpreted descriptively because heterogeneous indications and dosing precluded quantitative synthesis.

Pharmacology of sirolimus in pregnancy

The mTOR pathway integrates signals from nutrients, growth factors, and cellular stress to regulate protein translation, metabolism, and cell size [1,2]. mTOR forms two functionally distinct complexes: mTORC1, which is sensitive to sirolimus and regulates S6 kinase and 4E-BP1-dependent translation, and mTORC2, which contributes to AKT signaling and cytoskeletal function [1]. TSC1 and TSC2 encode hamartin and tuberin, which suppress mTORC1. Pathogenic variants in either gene lead to mTORC1 activation and hamartomatous growth, explaining the biological plausibility of mTOR inhibition in TSC-related fetal tumors [3,4].

Sirolimus is orally administered, extensively distributed, metabolized mainly by CYP3A4, and transported by P-glycoprotein. Drug interactions with CYP3A4 inhibitors or inducers are clinically important, and grapefruit products should be avoided. In transplantation, trough concentrations are individualized; in fetal therapy reports, maternal trough targets are generally extrapolated from pediatric and vascular anomaly practice rather than validated by fetal pharmacokinetic studies [5,9-18].

The pharmacological appeal of sirolimus in pregnancy is balanced by regulatory caution. Product labeling states that sirolimus can cause fetal harm based on animal studies, including embryo-fetal toxicity during organogenesis [23]. UK Teratology Information Service and transplant pregnancy sources indicate that human data are limited and do not show a consistent pattern of malformations. Still, they cannot exclude risk because exposed pregnancies are few and often confounded by maternal disease and concomitant medication [24-27]. Therefore, prenatal sirolimus should not be regarded as routine pregnancy medication; it is a fetal intervention justified only when the expected fetal benefit outweighs maternal and fetal uncertainty.

Placental transfer and fetal pharmacokinetics

Successful fetal pharmacotherapy requires placental transfer. Published prenatal cases demonstrate clinically meaningful fetal exposure because fetal tumors and malformations respond during ongoing maternal treatment [9-22]. Direct evidence is strongest in a fetal lymphatic malformation report in which repeated cordocentesis documented transplacental sirolimus passage of approximately 30% [20]. For cardiac rhabdomyomas, tumor shrinkage and improved fetal hemodynamics within weeks of therapy provide indirect but compelling evidence of biologically active fetal concentrations [9-19].

Most reported protocols initiate sirolimus after detailed counseling and then titrate the maternal dose to achieve trough concentrations. Published fetal cardiac rhabdomyoma cases have used regimens ranging from approximately 1 to 6 mg/day, with trough concentrations from below 1 ng/mL to approximately 12 ng/mL [9-19]. Vascular anomaly reports have commonly used maternal oral dosing in the late second or third trimester, with continuation to delivery or temporary discontinuation before planned delivery to reduce concerns about wound healing and infection [20-22].

No validated fetal trough target exists. Practical management, therefore, depends on triangulation among maternal trough level, maternal toxicity, serial ultrasound or echocardiographic response, gestational age, and urgency of delivery. When a lesion stabilizes and fetal compromise resolves, dose reduction or discontinuation can be considered; however, regrowth after discontinuation has been reported in the cardiac rhabdomyoma literature [18,19].

Fetal cardiac rhabdomyomas and tuberous sclerosis complex

Cardiac rhabdomyoma is the most common fetal cardiac tumor and is highly associated with TSC, particularly when lesions are multiple [28-32]. Many tumors are clinically silent and regress after birth; therefore, prenatal sirolimus is not indicated for every fetus with rhabdomyoma. The therapeutic question arises when tumor position or burden produces outflow obstruction, inflow obstruction, arrhythmia, impaired ventricular function, pericardial effusion, hydrops, polyhydramnios due to fetal cardiac compromise, or a credible risk of intrauterine demise [9-19].

The first major signal that prenatal mTOR inhibition could alter fetal outcome came from a 2018 report of maternal sirolimus therapy for fetal cardiac rhabdomyomas [9]. Subsequent cases and small series described rapid tumor regression, improved hemodynamics, and live birth after treatment for large or multiple tumors [10-18]. The literature includes reports by Park et al., Vachon-Marceau et al., Pluym et al., Dagge et al., Ebrahimi-Fakhari et al., Will et al., Goncalves et al., and Uno et al., among others [10-18]. A recent review and proposed management algorithm identified 13 studies involving 15 fetuses and reported that sirolimus was the most frequently used mTOR inhibitor; all included studies described tumor reduction and improved cardiac function, although regrowth after discontinuation occurred in some cases [18].

The roles of ultrasound and fetal echocardiography are decisive. Baseline assessment should document number, maximal diameter, location, relation to inflow and outflow tracts, valve function, ventricular performance, cardiothoracic ratio, rhythm, venous Doppler, hydrops, and associated extracardiac findings. Fetal MRI can support brain assessment in suspected TSC, and molecular testing of TSC1/TSC2 can inform diagnosis, counseling, and neonatal surveillance [3,4,28-32].

Treatment should be considered for symptomatic or threatening lesions, not for stable incidental tumors. Candidate fetuses include those with severe obstruction, declining function, arrhythmia attributable to tumor, hydrops, rapidly increasing tumor burden, or anticipated neonatal instability. In contrast, observation is appropriate for small, stable, non-obstructive tumors with normal rhythm and function. Because spontaneous regression may occur, every decision must explicitly compare expected natural history with the risks of maternal treatment (Table 1).

**Table 1:** Suggested monitoring during prenatal sirolimus therapy

Domain	Representative publications	Baseline	During treatment
Maternal laboratory	Adams et al., Freixo et al., Triana et al., Hammer et al. [5-8]; Pfizer-Info [23]; UK Med in Pregnancy [24]; Sifontis et al., Tshering et al., Armenti et al. [25-27]	CBC, renal function, liver enzymes, fasting lipids, urine protein as indicated	CBC, renal/liver function and lipids every 1-2 weeks initially; adapt to toxicity
Drug monitoring	Barnes et al., Park et al., Vachon-Marceau et al., Pluym et al., Dagge et al., Ebrahimi-Fakhari et al., Will et al., Goncalves et al., Uno et al. [9-17]; Vergote et al., Seront et al., Klosowska et al., Iacobas et al. [19-22]; Pfizer-Info [23]	Medication interaction review; establish target trough locally	Sirolimus trough after initiation and dose changes; avoid CYP3A4 interactions
Fetal imaging	Barnes et al., Park et al., Vachon-Marceau et al., Pluym et al., Dagge et al., Ebrahimi-Fakhari et al., Will et al., Goncalves et al., Uno et al. [9-17]; Vergote et al., Seront et al., Klosowska et al., Iacobas et al. [19-22]	Detailed ultrasound, fetal echocardiography, MRI when indicated	Weekly imaging in unstable cases; objective size and hemodynamic measurements
Delivery planning	Barnes et al., Park et al., Vachon-Marceau et al., Pluym et al., Dagge et al., Ebrahimi-Fakhari et al., Will et al., Goncalves et al., Uno et al. [9-17]; Vergote et al., Seront et al., Klosowska et al., Iacobas et al. [19-22]; Pfizer-Info [23]	Identify tertiary fetal/neonatal center	Coordinate timing, route, anesthesia, airway/cardiac/surgical teams, perioperative discontinuation when feasible

Fetal vascular and lymphatic malformations

Vascular anomalies are classified by the International Society for the Study of Vascular Anomalies (ISSVA) into vascular tumors and vascular malformations, with increasing integration of causal genes and signaling pathways [33-36]. Complex malformations may involve capillary, venous, lymphatic, and arteriovenous components, and some belong to the PIK3CA-related overgrowth spectrum. The PI3K-AKT-mTOR pathway is a common downstream node in several malformation syndromes, supporting the use of mTOR inhibition in selected lesions [33-38].

Postnatal experience provides an important background. The phase II trial by Adams et al. demonstrated clinical activity and acceptable tolerability of sirolimus in complicated vascular anomalies [5].

Lymphatic malformations are congenital, non-neoplastic anomalies caused by abnormal lymphatic development. They frequently involve the head and neck but may occur throughout the body. Ultrasound identifies cystic architecture and hemodynamic features, whereas magnetic resonance imaging defines the full anatomic extent and relationship to the airway and other vital structures. Management ranges from surveillance to sclerotherapy, surgery, or a combination of treatments. In extensive or anatomically complex disease, complete resection may be impossible, and morbidity may remain substantial; systemic targeted therapy with sirolimus has therefore expanded the therapeutic options.

Subsequent systematic reviews, series, and expert practice reports support sirolimus for severe lymphatic anomalies, venous/lymphatic malformations, and vascular tumors when morbidity is substantial and conventional options are inadequate [6-8,37-40].

Prenatal evidence is newer. Seront et al. reported early fetal management of a large cervicofacial lymphatic malformation with maternal sirolimus from 22 weeks' gestation until two weeks before planned delivery; cordocentesis demonstrated placental passage, and postnatal continuation allowed residual mass resection with long-term follow-up [20]. Klosowska et al. reported effective prenatal and postnatal sirolimus treatment in two extensive congenital capillary-lymphatic-venous malformations diagnosed by ultrasound and confirmed by MRI; maternal treatment began at 32 and 33 weeks, respectively, in pregnancies complicated by intralesional bleeding and polyhydramnios [21]. A 2025 Prenatal Diagnosis report described in-utero therapy for fetal vascular anomalies and emphasized that evidence remains limited but clinically important for large lymphatic malformations [22].

The prenatal indication differs from postnatal vascular anomaly treatment. Fetal therapy should be considered only when the lesion threatens survival or organ function before or immediately after birth. Examples include massive cervicofacial lesions threatening airway obstruction, lesions with intralesional hemorrhage, expanding thoracoabdominal lymphatic malformations causing hydrops, high-flow tumors with consumptive coagulopathy or high-output failure, and lesions likely to prevent safe delivery without prenatal stabilization. Fetal MRI complements ultrasound, particularly for airway, mediastinal, thoracic, and pelvic extension.

Maternal safety and monitoring

Maternal monitoring is mandatory. Baseline evaluation should include complete blood count, renal function, liver enzymes, fasting lipid profile, glucose if clinically indicated, urine protein assessment when relevant, infection history, vaccination status, medication review for CYP3A4 interactions, and surgical planning. During therapy, maternal sirolimus trough levels should be checked regularly and after dose changes. Complete blood count, liver enzymes, renal function, and lipids should be followed every one to two weeks initially, then individualized.

Common toxicities include oral ulcers or mucositis, hyperlipidemia, thrombocytopenia, leukopenia, anemia, edema, acneiform or eczematous eruptions, gastrointestinal symptoms, hypertension, proteinuria, impaired wound healing, and increased infection susceptibility [5-8,23]. Hypertriglyceridemia was highlighted in a fetal rhabdomyoma case and required dose adjustment [16]. Fetal growth restriction has been reported with maternal sirolimus therapy, although causality is difficult to establish because severe fetal disease, placental factors, and maternal comorbidity may contribute [26].

Peripartum management should be planned early. Because sirolimus may impair wound healing, many teams consider stopping therapy before planned cesarean delivery when fetal condition permits or reducing the dose if maternal toxicity develops. However, discontinuation may permit lesion regrowth; therefore, stopping rules must balance maternal surgical risk against fetal disease control [18-21]. Neonatology, pediatric cardiology, pediatric surgery, interventional radiology, genetics, and pharmacy should be involved before delivery (Table 2).

**Table 2:** Key evidence supporting prenatal sirolimus CLVM: capillary-lymphatic-venous malformation; UKTIS: UK Teratology Information Service

Evidence type	Representative publications	Main finding	Limitations
Fetal cardiac rhabdomyoma case reports/series	Barnes et al., Park et al., Vachon-Marceau et al., Pluym et al., Dagge et al., Ebrahimi-Fakhari et al., Will et al., Goncalves et al., Uno et al., Martinez-Garcia et al. [9-18]	Tumor regression and improved fetal hemodynamics in symptomatic cases	Small numbers, publication bias, heterogeneous dosing
Review/algorithm	Martinez-Garcia et al. [18]	Summarized 15 fetuses; proposed prenatal management algorithm	Narrative evidence, no randomized data
Fetal lymphatic/CLVM reports	Seront et al., Klosowska et al., Prenatal Diagnosis report [20-22]	Prenatal stabilization of selected severe lesions	Very small case numbers
Pregnancy safety data	Product labeling, UKTIS, transplant registry [23-27]	Animal embryo-fetal toxicity; no consistent human malformation signal in limited data	Not specific to fetal therapy; confounding by maternal disease

Fetal and neonatal outcomes

Reported fetal and neonatal outcomes are encouraging but should not be overinterpreted. In fetal cardiac rhabdomyoma, published cases generally show tumor shrinkage, improved fetal hemodynamics, and survival to delivery [9-19]. The 2025 review of fetal rhabdomyomas susceptible to prenatal mTOR inhibition found no fetal or neonatal deaths among 15 reported cases and no need for postnatal cardiac surgery at hospital discharge, but also emphasized the small sample size and risk of publication bias [18]. Neonatal management depends on the underlying disorder. Infants with TSC require brain MRI, EEG surveillance, dermatologic assessment, and a postnatal treatment pathway. Long-term data are sparse; the six-year follow-up after fetal lymphatic malformation treatment is reassuring but cannot define safety for all indications (Table 3) [20].

**Table 3:** Candidate fetal indications for prenatal sirolimus CLVM: capillary-lymphatic-venous malformation; TSC: tuberous sclerosis complex

Condition	Representative publications	High-risk fetal features	Assessment tools	Therapeutic goal
Cardiac rhabdomyoma/TSC	Barnes et al., Park et al., Vachon-Marceau et al., Pluym et al., Dagge et al., Ebrahimi-Fakhari et al., Will et al., Goncalves et al., Uno et al., Martinez-Garcia et al., Vergote et al. [9-19]; Mlczoch et al., Isaacs, Bader et al., Smythe et al., Jozwiak et al. [28-32]	Outflow obstruction, arrhythmia, ventricular dysfunction, hydrops, rapid growth	Fetal echocardiography, ultrasound, MRI/genetic testing	Tumor regression, hemodynamic stabilization, term or near-term delivery
Cervicofacial lymphatic malformation	Seront et al. [20]; Iacobas et al. [22]	Airway threat, rapid enlargement, polyhydramnios, hydrops	Ultrasound, MRI, airway planning	Stabilize lesion, permit controlled delivery and airway management
Complex CLVM/PROS-related lesion	Klosowska et al. [21]; ISSVA [33]; Goldberg et al., Kuimoto et al., Wassef et al., Wang et al., Boscolo et al., Luks et al. [33-39]	Intralesional bleeding, massive size, hydrops, coagulopathy risk	Ultrasound, MRI, hematologic evaluation	Reduce progression and perinatal instability
Vascular tumor with high-output or consumptive physiology	Adans et al., Freixo et al., Dore et al., Hammer et al. [5-8]; ISSVA, Goldenberg et al., Kunimoto et al., Wassef et al., Wang et al., Boscolo et al., Luks et al., Neirotti et al. [33-40]	Cardiac overload, thrombocytopenia/coagulopathy, fetal distress	Ultrasound, Doppler, fetal echocardiography, labs	Prevent fetal decompensation and allow neonatal therapy

Clinical treatment algorithm

Step 1

Confirm diagnosis and severity: Use expert ultrasound, fetal echocardiography, and fetal MRI when indicated. Define lesion type using ISSVA nomenclature for vascular anomalies and assess TSC probability for rhabdomyomas.

Step 2

Determine whether observation is sufficient: Stable non-obstructive rhabdomyomas and vascular anomalies without fetal compromise are usually managed expectantly with serial imaging.

Step 3

Identify high-risk features: (i) For rhabdomyoma, obstruction, arrhythmia, ventricular dysfunction, hydrops, rapid growth, or anticipated neonatal instability; (ii) for vascular anomalies, hydrops, intralesional bleeding, airway threat, high-output failure, consumptive coagulopathy, rapidly progressive size, or delivery-threatening anatomy.

Step 4

Convene a multidisciplinary fetal therapy board: Include maternal-fetal medicine, fetal cardiology, neonatology, genetics, pediatric surgery, vascular anomaly specialists, pediatric cardiology, anesthesia, pharmacy, and ethics when appropriate.

Step 5

Counsel parents: Discuss natural history, alternative management, uncertainties, animal embryo-fetal toxicity, limited human safety data, maternal adverse effects, potential fetal benefit, neonatal plans, and long-term follow-up.

Step 6

Initiate treatment only when expected benefit outweighs risk: Begin oral sirolimus with therapeutic drug monitoring. Avoid interacting medications and monitor maternal blood count, renal and liver function, and lipids.

Step 7

Assess response: Repeat ultrasound or fetal echocardiography at least weekly during the early response period in unstable cases, then individualize. Document objective changes in tumor or lesion size, Doppler, function, hydrops, and growth.

Step 8

Plan delivery: Decide timing and location based on fetal stability, airway or cardiac risk, maternal toxicity, and need for EXIT procedure, neonatal intensive care, postnatal imaging, surgery, sclerotherapy, or continuation of mTOR inhibition.

Step 9

Follow long-term: Record neonatal drug exposure, infection, wound healing, growth, neurodevelopment, TSC manifestations, vascular anomaly recurrence, and need for postnatal therapy.

Discussion and future directions

Prenatal sirolimus illustrates a broader transition in fetal medicine from procedure-based intervention to pathway-directed pharmacotherapy. The available literature is insufficient to define the standard of care. Still, it is sufficient to justify consideration in selected high-risk cases when managed by teams experienced in fetal diagnosis, maternal pharmacology, and neonatal rescue.

The most compelling indication is a symptomatic fetal cardiac rhabdomyoma associated with TSC. The biological rationale is direct, the imaging endpoints are measurable, and multiple independent reports describe tumor shrinkage after maternal therapy [9-19]. Nevertheless, the denominator of untreated fetuses is uncertain, spontaneous regression may occur, and published cases are likely enriched for successful outcomes. Future research should therefore define which fetuses require treatment and which can be safely observed.

For vascular and lymphatic malformations, the evidence is more preliminary but clinically important. Lesions threatening the airway, causing hydrops or bleeding, or creating a high-risk delivery scenario may benefit from stabilization before birth [20-22]. However, vascular anomalies are biologically heterogeneous. ISSVA classification, MRI phenotyping, and molecular diagnosis should be integrated wherever possible [33-36]. As targeted therapies expand, prenatal management may eventually differentiate between mTOR-sensitive lesions, PI3K-alpha-sensitive lesions, and lesions better approached by procedural or postnatal therapy.

Safety remains the central unresolved issue. Regulatory labeling is appropriately cautious because animal data show embryo-fetal toxicity [23]. Human pregnancy data do not show a consistent teratogenic pattern, but numbers remain small and are not derived from controlled fetal therapy trials [24-27]. Because most prenatal sirolimus use begins after organogenesis, the teratogenic risk may differ from that of first-trimester exposure; however, the effects on growth, the immune system, metabolism, and neurodevelopment remain uncertain. Long-term registries should be mandatory.

Imaging should be regarded as both a diagnostic method and a therapeutic monitoring tool. Serial ultrasound and fetal echocardiography should quantify disease trajectory before treatment and response after treatment. Reports should include standardized measurements, gestational age at treatment initiation and discontinuation, maternal dose and trough level, toxicity, delivery details, neonatal therapy, and follow-up. Without standardized reporting, the field will remain dependent on anecdotal evidence.

The future of prenatal sirolimus will depend on multicenter collaboration. Given the rarity of candidate conditions, randomized trials may be difficult to conduct, but prospective registries with predefined imaging endpoints, pharmacokinetic sampling, and long-term child outcomes are feasible. Integration of fetal genomics, placental pharmacology, and international vascular anomaly networks could transform prenatal sirolimus from a compassionate therapy to an evidence-based, targeted fetal treatment.

## Conclusions

Prenatal sirolimus is a promising but still investigational fetal therapy. Current evidence supports consideration for life-threatening or organ-threatening fetal cardiac rhabdomyomas, particularly in TSC, and for selected severe vascular or lymphatic malformations. Treatment should occur only after expert imaging, multidisciplinary review, and detailed parental counseling. Maternal trough monitoring, toxicity surveillance, serial fetal imaging, and structured neonatal follow-up are essential. The next stage of development should prioritize prospective registries, harmonized reporting, fetal pharmacokinetics, and long-term safety.
